# Expression signatures of long non-coding RNAs in early brain injury following experimental subarachnoid hemorrhage

**DOI:** 10.3892/mmr.2015.3474

**Published:** 2015-03-11

**Authors:** BINGJIE ZHENG, HUAILEI LIU, RUKE WANG, SHANCAI XU, YAOHUA LIU, KAIKAI WANG, XU HOU, CHEN SHEN, JIANING WU, XIN CHEN, PEI WU, GUANG ZHANG, ZHIYONG JI, HONGYU WANG, YAO XIAO, JIANYI HAN, HUAIZHANG SHI, SHIGUANG ZHAO

**Affiliations:** Department of Neurosurgery, The First Affiliated Hospital of Harbin Medical University, Harbin, Heilongjiang 150001, P.R. China

**Keywords:** long non-coding RNAs, subarachnoid hemorrhage, microarray, early brain injury

## Abstract

Subarachnoid hemorrhage (SAH) is an important cause of mortality in stroke patients. Long non-coding RNAs (LncRNAs) have important functions in brain disease, however their expression profiles in SAH remain to be elucidated. The present study aimed to investigate the expression signatures of LncRNAs and mRNAs in early brain injury (EBI) following SAH in a rat model. Male Wistar rats were randomly divided into an SAH group and a sham operation group. The expression signatures of the LncRNAs and mRNAs in the temporal lobe cortex were investigated using a rat LncRNAs array following experimental SAH. The results revealed that there were 144 downregulated and 64 upregulated LncRNAs and 181 downregulated and 221 upregulated mRNAs following SAH. Additionally, two upregulated (BC092207, MRuc008hvl) and three downregulated (XR_006756, MRAK038897, MRAK017168) LncRNAs were confirmed using reverse transcription quantitative polymerase chain reaction. The differentially expressed mRNAs were further analyzed using the Gene Ontology and the Kyoto Encyclopedia of Genes and Genomes (KEGG) databases. The pathway analysis results provided by the KEGG database indicated that eight pathways associated with inflammation were involved in EBI following SAH. In conclusion, these results demonstrated that the expression profiles of the LncRNAs and mRNAs were significantly different between the SAH-induced EBI group and the sham operation group. These differently expressed LncRNAs may be important in EBI following SAH.

## Introduction

Subarachnoid hemorrhage (SAH) is a type of stroke, which affects 1–12 individuals per 100,000 annually, worldwide. Despite advances in management strategies, the prognosis of SAH remains poor and the mortality rate has remained relatively unchanged (1). Accumulating evidence has indicated that early brain injury (EBI) is one of the leading causes of mortality worldwide in patients with SAH (2). However, the mechanism underlying the early pathophysiological consequences of SAH, and how to alleviate them, remain to be elucidated. Previous studies have revealed that certain pathways and genes associated with inflammation are involved in these pathophysiological processes and affect cell apoptosis or necrosis following SAH; therefore, targeting these pathways or genes can significantly ameliorate brain injury (3–8). Additionally, with developments of genomics, numerous genes have been reported to be differentially expressed in SAH (9,10). However, the molecular regulatory mechanism in EBI following SAH remains to be fully elucidated.

Long non-coding R NAs (LncR NAs), which contain >200 nucleotides, have been demonstrated to regulate the biological behavior of cells (11–13). Previous studies have revealed that LncRNAs are involved in various pathophysiological processes in brain disease and that targeting LncRNAs effectively reverses the progress of ischemic stroke, Alzheimer’s disease and brain tumors (14–17). LncRNAs have specific expression profiles in brain tissues and are potential independent prognostic molecular markers (18). There is evidence to suggest that LncRNAs may be important for regulating the process of brain disease (14–18). However, the expression and function of LncRNAs in EBI following SAH remain to be elucidated. Thus, the present study aimed to investigate whether differently expressed LncRNAs are important in the early pathophysiological stage following SAH.

The present study established a rat SAH model, as previously reported (2,19) and used microarray assays to identify the expression profiles of LncRNAs and mRNAs in tissues of the temporal lobe cortex 24 h after SAH. Thie five differentially expressed LncRNAs identified were further confirmed in additional SAH samples via reverse transcription quantitative polymerase chain reaction (RT-qPCR). In addition, differentially expressed mRNAs were analyzed using the Gene Ontology (GO) and the Kyoto Encyclopedia of Genes and Genomes (KEGG) databases to clarify their biological functions. Therefore, the present study aimed to investigate the LncRNA and mRNA expression profiles and functions in EBI following SAH.

## Materials and methods

### Animal model and sample preparation

Adult male Wistar rats (250–300 g; n=16) were purchased from Yisi Laboratory Animal Technology Co., Ltd. (Changchun, China). The rats were housed in a room under an alternating 12-h light/dark cycle, with a controlled temperature (22–24°C) and humidity of ~55%. The rats were anesthetized with 10 % chloral hydrate (0.3 ml/100 g body weight; Hubei Jusheng Technology Co., Ltd, Tianmen, China). Non-heparinized fresh autologous arterial blood (0.3 ml) was slowly injected into the pre-chias-matic cistern for 25 sec using a syringe (Jiangxi Hongda Medical Equipment Group, Ltd., Jiangxi, China). Control animals were injected with 0.3 ml saline (Harbin Pharmaceutical Group Co., Ltd, Shanghai, China). Images of the rats brains were captured using a Canon IXY 31S camera (Canon, Inc., Tokyo, Japan) under the following conditions: Aperture value, f/2; exposure time, 1/20 sec; and focal distance, 4 mm. The temporal lobe cortex was dissected 24 h following SAH and the brain samples were rapidly frozen with liquid nitrogen (Harbin, Liming Gas Group, Co., Ltd., Harbin, China) and then stored at -80°C until further use. The present study was approved by the Ethical Committee of the First Affiliated Hospital of Harbin Medical University (Harbin, China).

### RNA extraction and quality control

The total RNA was extracted from three-paired samples from the SAH and control groups using TRIzol reagent (Invitrogen Life Technologies, Carlsbad, CA, USA) according to the manufacturer’s instructions. The quantification and quality of the RNA was measured using a NanoDrop ND-1000 spectrophotometer (NanoDrop Technologies, Inc., Wilmington, DE, USA). The integrity of the RNA and DNA contamination were assessed using denaturing agarose gel electrophoresis (Invitrogen Life Technologies). The gels were prepared with agarose (111860; Beijing Borunlaite Science & Technology Co., Ltd. Beijing, China), 10XMOPS running buffer [M00138; Genscript (Nanjing) Co., Ltd., Nanjing, China] and 37% formaldehyde (M134–500ML; Shanghai Haoran Bio-Technology Co., Ltd., Shanghai, China). Subsequently, six wells were prepared with the 10XMOPS running buffer. Finally, the prepared samples were loaded into the wells, electrophoresis was conducted and then the gels were visualized using a UV transilluminator (170–8170; Bio-Rad Laboratories, Inc., Hercules, CA, USA).

### Array hybridization and data analysis

Preparation of the three-paired samples and microarray assays were performed using the Agilent array platform (Agilent Technologies, Inc., Santa Clara, CA, USA) according to the manufacturer’s instructions, with modifications. Briefly, the mRNAs were amplified and transcribed into fluorescent complementary (c)RNAs using a random priming method, according to Agilent’s Quick Amp Labeling instructions (version 5.7; Agilent Technologies, Inc.). The cRNAs were purified using an RNeasy Mini kit (Qiagen, Hilden, Germany) and mixed with Agilent Gene Expression Hybridization Kit (p/n 5188–5242; Agilent Technologies, Inc.) containg 10X blocking agent, 25X fragmentation buffer, 2X GEx hybridization buffer HI-RPM. The mixture was then transferred to the rat LncRNs array v2.0 expression slide (4×44K; Arraystar, Shanghai, China). Following washing and fixing using Gene Expression Buffer 1 (p/n, 5188–5325; Agilent Technologies, Inc.) and Buffer 2 (p/n, 5188–5326; Agilent Technologies, Inc.), respectively, the arrays were scanned using an Agilent DNA Microarray Scanner (G2505C; Agilent Technologies, Inc.). Images were acquired and were analyzed using Agilent Feature Extraction software (version 11.0.1.1; Agilent Technologies, Inc.) and subsequent data processing was performed using GeneSpring GX v11.5.1 software (Agilent Technologies, Inc.). The data were filtered using a Volcano plot and then differentially expressed (>2.0 fold; P<0.05) LncRNAs and mRNAs were assessed by constructing a box plot and scatter plot, respectively, to ensure the quality of the data. Based on the expression levels (log_2_ ratio) of the differentially expressed mRNAs (>3.0 fold change; P<0.05) and lncRNAs (>3.0 fold change; P<0.05), the two-way hierarchical clustering were carried out using the R package ‘pheatmap’, version 0.7.7 (http://cran.r-project.org/web/packages/pheatmap/). The microarray data were prepared and analyzed by Oebiotech Co., Ltd. (Shanghai, China).

### GO and KEGG pathway analysis

The differentially expressed mRNAs were selected for GO and KEGG pathway analysis. The GO project describes gene attributes, including biological process (BP), cellular component (CC) and molecular function (MF). Based on the GO categories (http://www.geneontology. org), the differentially expressed mRNAs were classified under different GO terms according to their characteristics and the enrichment of the GO terms was calculated. The KEGG database (http://www.genome.jp/kegg) was used to analyze the differentially expressed mRNAs and the enrichment of different pathways was also calculated. The P-value indicated the significatnce of the GO term and KEGG pathway enrichment (P<0.05). The false discovery rate (FDR) was used to evaluate the significance of the P-value and an FDR<0.05 was recommended. The GO and KEGG pathway analyses were performed by Oebiotech Co., Ltd. (Shanghai, China).

### RT-qPCR validation

The total RNA was extracted from the SAH and control groups (five-paired) using TRIzol reagent (Invitrogen Life Technologies). In order to validate the reliability of the microarray data, five randomly selected lncRNAs and their expression levels were further assed by RT-qPCR using a SYBR Green PCR Master mix kit (Applied Biosystems Life Technologies, Foster City, CA, USA) on an ABI 7500HT Fast Real-Time PCR instrument (Applied Biosystems Life Technologies). The raw data were normalized to the expression of actin. The following primers were used: MRuc008hvl, forward 5′-GGACATCCAGATGCTGTT-3′ and reverse 5′-ACTGATGGTTTGCTCCATTA-3′; MRAK038897, forward 5′-TGCTGAAGACCAATGAGTTT-3′ and reverse 5′-TCTGACTTGTGATCTACAGGC-3′; BC092207, forward 5′-TAAGCTGTAATCTACGGGAGG-3′ and reverse 5′-GCT GTTTCATCAGGTTGTCATA-3′; MRAK017168, forward 5′-TTACCTGGAACTGTACCCTCT-3′ and reverse 5′-CTC CTCCTAGCCATCTCAAT-3′; XR_006756, forward 5′-ACT GGTAACCTCCTGCTC-3′ and reverse 5′-TGGTGGCTC GTCTACTT-3′ and actin, forward 5′-CCCATCTATGAG GGTTACG-3′ and reverse 5′-ATGTCACGCACGATTTCC-3′.

### Statistical analysis

Student’s t-test was used to evaluate the differences in the expression levels of LncRNAs between the SAH and control groups using Prism5 statistical software (GraphPad Software, Inc., La Jolla, CA, USA). P<0.05 was considered to indicate a statistically significant difference.

## Results

### Quality assessment of the LncRNA and mRNA data between the SAH model and the control group

**Figure 1:**
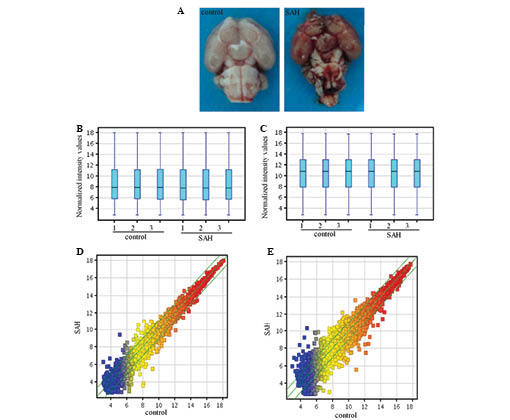
Quality assessment of the expression levels of LncRNAs and mRNAs in the SAH model and control group. (A) Representative images of the rat brain 24 h after SAH or in the sham operation control. Box-plots (data normalised using Log2) were used to evaluate the quality of the expression data of (B) LncRNAs and (C) mRNAs. Scatter-plots (data normalised using Log2) were used to evaluate the variation in the expression of (D) LncRNAs and (E) mRNAs between the SAH model and control group. The distributions of the intensities were normalized by log_2_-ratios and presented in a scatter-plot and box plot, respectively. The values of the X and Y axes in the scatter plot are the averaged normalized signal values of the group (log_2_ scaled) The green lines represent the fold change (fold change=2.0). Values are presented as the mean ± standard error of the mean (n=3), experiments were performed in duplicate. SAH, subarachnoid hemorrhage; LncRNAs, long non-coding RNAs.

To investigate the expression of LncRNAs in SAH, a rat SAH model mimicking human SAH was established, as previously described (2,19). As demonstrated in Fig. 1A, the temporal lobe cortex of the SAH model and the control rats were resected and images were captured 24 h after resection. It was evident that the blood was present in the subarachnoid space of the SAH brain tissues. Microarray hybridization was performed to detect the expression profiles of the LncRNAs and mRNAs in the two groups. To ensure the quality of the microarray data, the microarray results were evaluated using box-plot and scatter-plot statistical methods. As indicated in Figs. 1B–E, the box-plot results demonstrated that the distributions of the intensities from all the samples were almost identical. Additionally, scatter-plot analysis indicated that the distribution of the LncRNA and mRNA profiles varied and were different between the two groups. In conclusion, these results demonstrated that the data exhibited a Gaussian distribution and were homogeneous.

### Differentially expressed LncRNAs and mRNAs between the two groups

**Figure 2:**
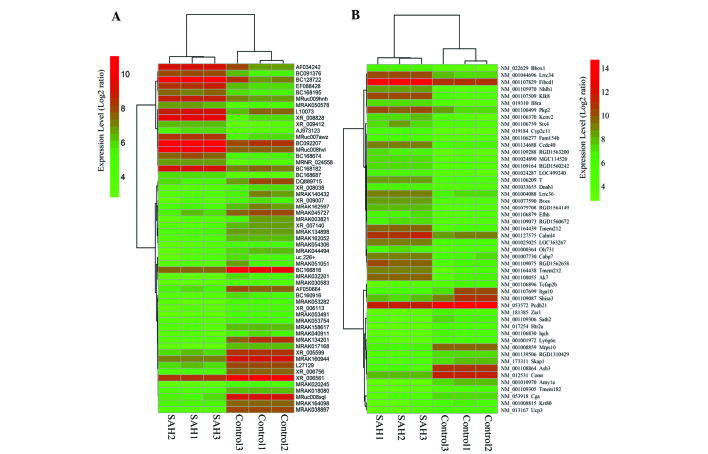
Differential expression profiles of the LncRNAs and mRNAs between the SAH model and control group. Heat maps revealed the expression profiles of the (A) LncRNAs and (B) mRNAs between the SAH model and control group (>3.0-fold change; P<0.05). Every sample was analyzed in triplicate. Green represents downregulated genes and red represents upregulated genes. SAH, subarachnoid hemorrhage; LncRNAs, long non-coding RNAs.

Statistically significant differentially expressed LncRNAs and mRNAs were identified through volcano plot filtering. There were ~221 upregulated and 181 downregulated mRNAs (fold change >2.0; P<0.05) and 64 upregulated and 144 downregulated and LncRNAs in the SAH brain tissues compared with the control group (>2.0-fold change; P<0.05). In order to further examine these differentially expressed genes, any genes which changed ≥3-fold were selected to construct a hierarchical clustering map. As shown in Fig. 2A and B, in these genes (>3.0 fold change; P<0.05), the number of upregulated LncRNAs was less than the number of downregulated LncRNAs, while the opposite effect was observed in the mRNAs. Notably, these results demonstrated that MRAK038897 was the LncRNA exhibiting the most marked change (21.8-fold change; P<0.01). MRAK038897 is associated with ankyrin repeat and suppressor of cyto-kines signalling box 3 (ASB3), which is involved in the neuronal inflammatory process of EBI (20–22). Therefore, MRAK038897 may be a vital factor in the regulation of EBI. In conclusion, these results revealed that the LncRNAs and mRNAs had different expression profiles in the SAH group compared with the control group.

### Validation of the expression levels of the LncRNAs using RT-qPCR

**Figure 3:**
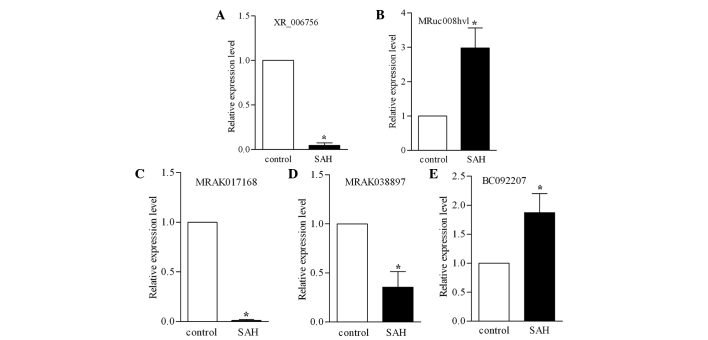
RT-qPCR validation. RT-qPCR was used to confirm the expression of five long non-coding RNAs, (A) XR_006756, (B) MRuc008hvl, (C) MRAK017168, (D) MRAK038897 and (E) BC092207, in the SAH model and control groups. Actin was used as an internal standard (^*^P<0.05). Values are presented as the mean ± standard error of the mean. SAH, subarachnoid hemorrhage; RT-qPCR, reverse transcription quantitative polymerase chain reaction.

To validate the microarray data, two upregulated (BC092207, MRuc008hvl) and three downregulated (XR_006756, MRAK038897 and MRAK017168) LncRNAs, were randomly selected and their expression levels were further examined using RT-qPCR. The CT values were normalized to actin. As shown in Figs. 3A–E, the selected LncRNAs were confirmed to be differentially expressed in the SAH model compared with the control group. Consistent with the microarray results, the expression levels of BC092207 and MRuc008hvl were significantly upregulated and the expression levels of XR_006756, MRAK038897 and MRAK017168 were markedly downregulated. Therefore, the microarray results were stable and consistent with the RT-qPCR data.

### GO and KEGG analysis of differentially expressed mRNAs

**Figure 4:**
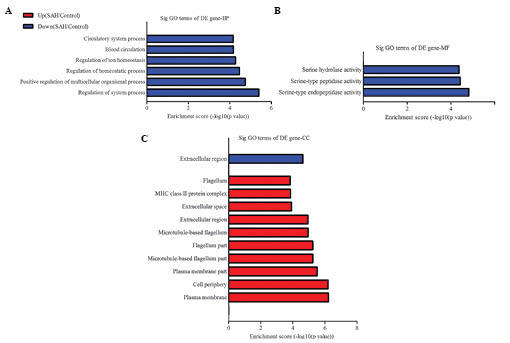
mRNAs that were differentially expressed were functionally classificated via GO analysis. The most enriched GO terms, which satisfied the P<0.05 and FDR<0.05 criteria. The downregulated or upregulated mRNAs were classified by (A) BP, (B) MF and (C) CC GO terms in the SAH model compared with the control group. SAH, subarachnoid hemorrhage, GO, Gene Ontology, DE, BP, biological processes; CC, cellular component; MF, molecular function; FDR, false discovery rate; DE, differentially expressed; up, upregulated; down, downregulated.

To clarify the potential function of the mRNAs on the regulation of the pathological process of SAH, GO categories were used to describe the BP, CC and MF of the differentially expressed mRNAs between the SAH group and the control group, as previously reported (23). The P-value (<0.05) and the FDR (<0.05) of the listed GO terms were used to evaluate the significance of the GO term enrichment in the differentially expressed mRNAs in the SAH group compared with the control groups. As shown in Fig. 4A–C, the GO analysis revealed that the ‘regulation of system process’, ‘serine-type endopeptidase activity’ and the ‘extracellular region’ were the enriched GO terms containing differentially downregulated mRNAs belonging to BP, MF and CC, respectively. The ‘plasma membrane’ was the most enriched GO term containing differentially unregulated mRNAs belonging to CC. In addition, the KEGG pathway analysis demonstrated that the ‘neuroactive ligand-receptor interaction’ and ‘leishmaniasis’ were the most enriched pathways containing differentially downregulated or upregulated mRNAs, respectively (Table I).

## Discussion

SAH is considered to be an important cause of mortality in stroke patients (1) and EBI is an important characteristic of SAH. Early clinical intervention can significantly reduce the incidence of sequelae and rate of mortality (2), therefore, identifying genes that affect the biological process of EBI is essential for the treatment of SAH. Although LncRNAs have been identified as important in the regulation of the pathophysiological process of brain disease (24–26), the expression profiles of LncRNAs in EBI following SAH remain to be elucidated. The present study investigated the expression profiles of LncRNAs in a rat SAH model and demonstrated that the expression levels of a number of LncRNAs were either upregulated or downregulated compared with the control group. Due to the abundance and specific expression of LncRNAs in different regions of the brain (18), these differentially expressed LncRNAs may be potential disease markers and key therapeutic targets.

According to the distribution of LncRNAs and splicing forms in the chromosome, LncRNAs are divided into sense, antisense, intronic, intergenic and bidirectional (10,27). The present study revealed that the majority of the LncRNAs belonged to these four categories. Additionally, several of the differently expressed LncRNAs exhibited multiple splicing forms and were correlated with multiple genes, including MRuc009bks, which was associated with five genes (Zc3hav1, Ttc26, RGD1310722, Fmc1 and Luc7l2). These results indicated that the transcription and regulatory mechanisms of LncRNAs are complex and require further investigation. Previous studies have indicated that the regulatory mechanisms of EBI correlate with multiple genes and are highly complex (2). The identification of these differentially expressed LncRNAs may provide further understanding of the mechanism underlying these complex biological regulatory processes.

In order to better understand these differentially expressed mRNA genes, the KEGG and GO databases were used to analyze their potential biological functions. The results revealed that these differentially expressed mRNA genes were involved in eight pathways, including ‘leishmaniasis’, ‘calcium signaling’, ‘tuberculosis’, ‘asthma’, ‘*Staphylococcus aureus* infection’, ‘chemical carcinogenesis’, ‘antigen processing and presentation’ and ‘neuroactive ligand-receptor interaction’. These pathways were mainly associated with inflammation, which has been confirmed to result in dysfunction of the blood brain barrier and brain edema (28). Notably, the ‘neuroactive ligand-receptor interaction’ term contained genes, including cortistatin, which is associated with neuronal apoptosis and neurodegenerative disease (29,30). In addition, the ‘leishmaniasis pathway’, including iNOS, which is involved in the SAH process and significantly inhibits its activity has been found to ameliorate cerebral vasospasm following SAH (8,31).

In conclusion, the present study was the first, to the best of our knowledge, to investigate the expression profiles of LncRNAs in SAH. The results demonstrated that the expression levels of LncRNAs in SAH were significantly different compared with those in the control group, suggesting that these LncRNAs may be important in the pathophysiological process of SAH. However, the rat SAH model used was only partially able to simulate a ruptured cerebral aneurysm. In addition, LncRNAs have temporally and spatially differential expression in the brain and the present study only examined the expression of LncRNAs in the temporal lobe cortex 24 h after SAH. Therefore, further studies are required to describe and confirm the expression of LncRNAs in the brain following SAH. LncRNAs may become potential future biological targets and prognostic indicators for SAH.

## Figures and Tables

**Table I:** Most enriched pathways associated with inflammation in early brain injury following subarachnoid hemorrhage via Kyoto Encyclopedia of Genes and Genomes analysis.

Definition	Fisher-P-value	False discovery rate	Genes
Neuroactive ligand-receptor interaction	1.88914E-05	0.004892872	AGTR1A, CGA, CORT, EDNRB, GCG, GIPR, GRM4, HTR1D HTR2A, PLG, SCTR, TAC1, THRA
Leishmaniasis	6.414E-06	0.001661226	C3, PTGS2, RT1-BA, RT1-BB, RT1-DA, TGFB3
Calcium signaling pathway	2.71687E-05	0.003518345	ADRA1D, CAMK2D, GNA14 ORAI1, P2RX2, P2RX6, PHKG1, PTGER3, RYR2, TRHR
Tuberculosis	0.000118804	0.009356411	C3, CAMK2D, CD74, NFYA, RT1-BA, RT1-BB, RT1-DA, TGFB3
Asthma	0.000177772	0.009356411	RT1-BA, RT1-BB, RT1-DA
*Staphylococcus aureus* infection	0.000180626	0.009356411	C3, RT1-BA, RT1-BB, RT1-DA
Chemical carcinogenesis	0.000316345	0.01365554	CYP2C11, CYP2E1, GSTM2, GSTM6, PTGS2, SULT1A1
Antigen processing and presentation	0.000428187	0.01584293	CD74, NFYA, RT1-BA, RT1-BB, RT1-DA

## References

[1] Cahill J, Zhang JH (2009). Subarachnoid hemorrhage: is it time for a new direction?. Stroke.

[2] Sehba FA, Hou J, Pluta RM, Zhang JH (2012). The importance of early brain injury after subarachnoid hemorrhage. Prog Neurobiol.

[3] Aladag MA, Turkoz Y, Parlakpinar H, Ozen H, Egri M, Unal SC (2009). Melatonin ameliorates cerebral vasospasm after experimental subarachnoidal haemorrhage correcting imbalance of nitric oxide levels in rats. Neurochem Res.

[4] Cahill J, Calvert JW, Solaroglu I, Zhang JH (2006). Vasospasm and p53-induced apoptosis in an experimental model of subarachnoid hemorrhage. Stroke.

[5] Cahill J, Calvert JW, Marcantonio S, Zhang JH (2007). p53 may play an orchestrating role in apoptotic cell death after experimental subarachnoid hemorrhage. Neurosurgery.

[6] Yan J, Chen C, Hu Q (2008). The role of p53 in brain edema after 24 h of experimental subarachnoid hemorrhage in a rat model. Exp Neurol.

[7] Zhou C, Yamaguchi M, Colohan AR, Zhang JH (2005). Role of p53 and apoptosis in cerebral vasospasm after experimental subarachnoid hemorrhage. J Cereb Blood Flow Metab.

[8] Zheng B, Zheng T, Wang L, Chen X, Shi C, Zhao S (2010). Aminoguanidine inhibition of iNOS activity ameliorates cerebral vasospasm after subarachnoid hemorrhage in rabbits via restoration of dysfunctional endothelial cells. J Neurol Sci.

[9] Vikman P, Beg S, Khurana TS, Hansen-Schwartz J, Edvinsson L (2006). Gene expression and molecular changes in cerebral arteries following subarachnoid hemorrhage in the rat. J Neurosurg.

[10] Kapranov P, Cheng J, Dike S (2007). RNA maps reveal new RNA classes and a possible function for pervasive transcription. Science.

[11] Chu C, Qu K, Zhong FL, Artandi SE, Chang HY (2011). Genomic maps of long noncoding RNA occupancy reveal principles of RNA-chromatin interactions. Mol Cell.

[12] Ling H, Fabbri M, Calin GA (2013). MicroRNAs and other non-coding RNAs as targets for anticancer drug development. Nat Rev Drug Discov.

[13] Yang L, Lin C, Jin C (2013). LncRNA-dependent mechanisms of androgen-receptor-regulated gene activation programs. Nature.

[14] Dharap A, Nakka VP, Vemuganti R (2012). Effect of focal ischemia on long noncoding RNAs. Stroke.

[15] Sun Y, Wang Z, Zhou D (2013). Long non-coding RNAs as potential biomarkers and therapeutic targets for gliomas. Med Hypotheses.

[16] Tan L, Yu JT, Hu N (2013). Non-coding RNAs in Alzheimer’s disease. Mol Neurobiol.

[17] Wu P, Zuo X, Deng H, Liu X, Liu L, Ji A (2013). Roles of long noncoding RNAs in brain development, functional diversification and neurodegenerative diseases. Brain Res Bull.

[18] Mercer TR, Dinger ME, Sunkin SM, Mehler MF, Mattick JS (2008). Specific expression of long noncoding RNAs in the mouse brain. Proc Natl Acad Sci USA.

[19] Zhang J, Zhu Y, Zhou D, Wang Z, Chen G (2010). Recombinant human erythropoietin (rhEPO) alleviates early brain injury following subarachnoid hemorrhage in rats: possible involvement of Nrf2-ARE pathway. Cytokine.

[20] Chou SH, Feske SK, Atherton J (2012). Early elevation of serum tumor necrosis factor-alpha is associated with poor outcome in subarachnoid hemorrhage. J Investig Med.

[21] Jiang Y, Liu DW, Han XY (2012). Neuroprotective effects of anti-tumor necrosis factor-alpha antibody on apoptosis following subarachnoid hemorrhage in a rat model. J Clin Neurosci.

[22] Sozen T, Tsuchiyama R, Hasegawa Y (2009). Role of interleukin-1beta in early brain injury after subarachnoid hemorrhage in mice. Stroke.

[23] Li JP, Liu LH, Li J (2013). Microarray expression profile of long noncoding RNAs in human osteosarcoma. Biochem Biophys Res Commun.

[24] Ng SY, Lin L, Soh BS, Stanton LW (2013). Long noncoding RNAs in development and disease of the central nervous system. Trends Genet.

[25] Qureshi IA, Mattick JS, Mehler MF (2010). Long non-coding RNAs in nervous system function and disease. Brain Res.

[26] Qureshi IA, Mehler MF (2013). Long Non-coding RNAs: Novel targets for nervous system disease diagnosis and therapy. Neurotherapeutics.

[27] Huang Y, Liu N, Wang JP (2012). Regulatory long non-coding RNA and its functions. J Physiol Biochem.

[28] Ostrowski RP, Colohan AR, Zhang JH (2006). Molecular mechanisms of early brain injury after subarachnoid hemorrhage. Neurol Res.

[29] Diniz L, dos Santos TB, Britto LR (2013). Effects of chronic treatment with corticosterone and imipramine on fos immunoreactivity and adult hippocampal neurogenesis. Behav Brain Res.

[30] Zhang H, Liu B, Wu J (2012). Icariin inhibits corticosterone-induced apoptosis in hypothalamic neurons via the PI3-K/Akt signaling pathway. Mol Med Rep.

[31] O’Connor DM, O’Brien T (2009). Nitric oxide synthase gene therapy: Progress and prospects. Expert Opin Biol Ther.

